# Transcatheter Arterial Embolization (TAE) of Cancer-Related Bleeding

**DOI:** 10.3390/medicina59071323

**Published:** 2023-07-18

**Authors:** Roberto Minici, Giuseppe Guzzardi, Massimo Venturini, Federico Fontana, Andrea Coppola, Marco Spinetta, Filippo Piacentino, Armando Pingitore, Raffaele Serra, Davide Costa, Nicola Ielapi, Pasquale Guerriero, Biagio Apollonio, Rita Santoro, MGJR Research Team, Luca Brunese, Domenico Laganà

**Affiliations:** 1 Radiology Unit, Dulbecco University Hospital, 88100 Catanzaro, Italy; miniciroberto@gmail.com (R.M.); armando.pingitore@gmail.com (A.P.);; 2 Radiology Unit, Maggiore della Carità University Hospital, 28100 Novara, Italy; marcospinetta90@gmail.com; 3 Diagnostic and Interventional Radiology Unit, ASST Settelaghi, Insubria University, 21100 Varese, Italy; massimo.venturini@uninsubria.it (M.V.); federico.fontana@uninsubria.it (F.F.); andrea.coppola@asst-settelaghi.it (A.C.); filippo.piacentino@asst-settelaghi.it (F.P.); 4 School of Medicine and Surgery, Insubria University, 21100 Varese, Italy; 5 Vascular Surgery Unit, Department of Medical and Surgical Sciences, Magna Graecia University of Catanzaro, Dulbecco University Hospital, 88100 Catanzaro, Italy; rserra@unicz.it; 6 Department of Law, Economics and Sociology, Magna Graecia University of Catanzaro, 88100 Catanzaro, Italy; davide.costa@unicz.it; 7 Department of Public Health and Infectious Disease, Sapienza University of Rome, 00185 Rome, Italy; nicola.ielapi@uniroma1.it; 8 Radiology Unit, Santobono-Pausilipon Hospital, 80129 Naples, Italy; pasqualeguerriero@gmail.com; 9 Department of Medicine and Health Sciences, University of Molise, 86100 Campobasso, Italy; luca.brunese@unimol.it; 10 Radiology Unit, San Timoteo Hospital, 86039 Termoli, Italy; bapollonio@sirm.org; 11 Haemophilia and Thrombosis Center, Dulbecco University Hospital, 88100 Catanzaro, Italy; ritacarlottasantoro@gmail.com; 12 Magna Graecia Junior Radiologists Research Team, 88100 Catanzaro, Italy; radiologyumg@gmail.com; 13 Scientific Committee of the Italian National Institute of Health (Istituto Superiore di Sanità, ISS), 00161 Rome, Italy; 14 Department of Experimental and Clinical Medicine, Magna Graecia University of Catanzaro, 88100 Catanzaro, Italy

**Keywords:** cancer, bleeding, TAE, embolization, hemorrhage, tumor, embolic agents, endovascular

## Abstract

*Background and Objectives*: Roughly 10% of cancer patients experience an episode of bleeding. The bleeding severity can range from occasional trivial bleeds to major bleeding. The treatment for the bleeding may vary, depending on the clinical condition and anatomical site, and may include various strategies, among which TAE is a cornerstone of major bleeding management. However, the existing literature on tumor hemorrhages is inconsistent. The objective of this multicenter retrospective cohort study was to evaluate the effectiveness and safety of arterial embolization in the treatment of tumor hemorrhages in patients with solid cancers. *Materials and Methods*: The data for patients with solid cancers undergoing TAE for the management of tumor hemorrhages from January 2020 to May 2023 were gathered. *Results*: A total of 92 patients with cancer-related bleeding were treated between January 2020 and May 2023. No bleeding was detected by X-ray angiography (XA) in 12 (13%) cases; therefore, a blind embolization was performed. The most common bleeding site was the liver (21.7%). A total of 66 tumor hemorrhages were spontaneous. The most commonly used embolic agent was polyvinyl alcohol (PVA) particles (30.4%). Technical success was achieved in 82 (89.1%) cases, with an 84.8% clinical success rate related to 14 cases of rebleeding. Proximal embolization was performed for 19 (20.7%) patients. Complications were recorded for 10 (10.9%) patients. The 30-day bleeding-related mortality was 15.2%. The technical success, clinical success, proximal embolization rate, and 30-day rebleeding were worse in the subset of patients undergoing TAE with coils. *Conclusions*: Transcatheter arterial embolization (TAE) represents a viable and potentially life-saving therapeutic approach in the management of tumor hemorrhages, demonstrating a notable effectiveness and safety. The TAE of bleeding tumors using coils resulted in a higher rate of non-superselective proximal embolization, with a trend toward lower clinical success rates and higher rebleeding episodes.

## 1. Introduction

Roughly 10% of cancer patients experience an episode of bleeding [1]. The bleeding severity can range from occasional trivial bleeds to chronic occult anemia, and from low-volume oozing to major bleeding, in some cases catastrophic [1,2]. In 2005, the Scientific and Standardization Committee of the International Society on Thrombosis and Haemostasis introduced a new definition for major bleeding in non-surgical studies [3], which has been implemented by the European Medicines Agency (EMEA) [4]. Major bleeding is particularly distressing to patients, requires hospitalization, often prevents the continuation of chemotherapy and, in some cases, results in patient death [5,6,7].

Bleeding in cancer patients can be directly caused by the cancer itself, or by related medical conditions. The cancer itself can be the source of the bleeding through three main mechanisms. The first is spontaneous bleeding, caused by the local tumor invasion of vessels and tissues, or by the rupture of abnormal intratumoral vessels. The second mechanism is represented by iatrogenic factors, such as medical or interventional treatments (e.g., radiotherapy, bevacizumab, endoscopic procedures, biopsies, etc.). The third, rare cause of bleeding is from traumas resulting in tumor rupture [1,2,8,9]. Finally, bleeding can also be caused by cancer-related medical conditions, such as cancer-related thrombocytopenia, liver failure, and anticoagulant therapy, and may occur in anatomical regions other than the site of the tumor growth. The latter mechanism is more frequent in hematologic malignancies and advanced cancers [1,2,6,10].

The treatment of major bleeding may vary according to the clinical condition and anatomical site, and may include a supportive conservative medical strategy, radiotherapy, interventional radiology, or surgery [1,5,9,11]. Among the interventional radiology options, transcatheter arterial embolization (TAE) plays a pivotal role in the management of clinically significant hemorrhages [12,13,14]. However, the existing literature on tumor hemorrhages is inconsistent, and is often limited to small case series on a specific type of cancer [9,11,15,16,17]. In order to determine the effectiveness and safety of TAE for the treatment of tumor hemorrhages in patients with solid malignancies, a multicenter retrospective cohort study was conducted.

## 2. Materials and Methods

### 2.1. Study Design

**Figure 1:**
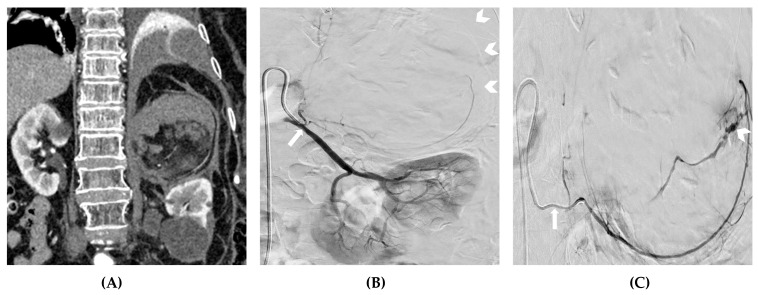

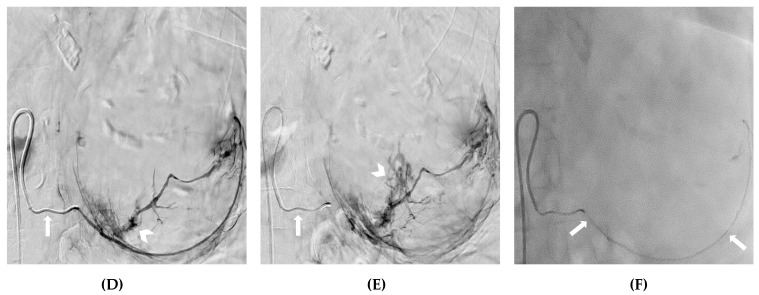
(**A**) A CTA showing spontaneous retroperitoneal bleeding due to an unknown, ruptured, adrenal myelolipoma. (**B**) A digital subtraction angiography depicting the inferior adrenal artery arising (arrow) from the proximal left renal artery, along with the shadow (arrowheads) of the ruptured myelolipoma displacing the left kidney downwards. (**C**,**D**,**E**) The superselective catheterization of the target artery with a microcatheter (arrow) and digital subtraction angiography, showing a massive intratumoral hemorrhage (arrowheads). (**F**) A fluoroscopy demonstrating the embolization of the inferior adrenal artery, using PVA particles mixed with iodinated contrast media (arrows).

**Figure 2:**
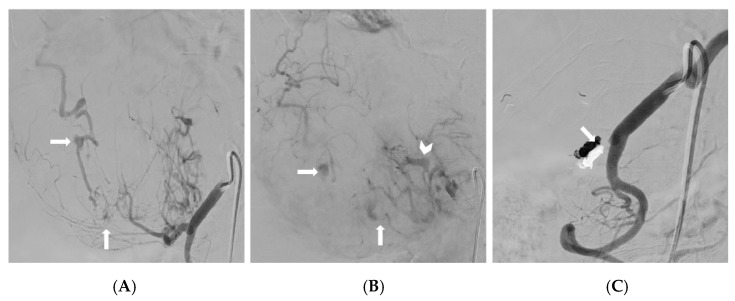
(**A**,**B**) The selective catheterization of the common hepatic artery, and the subsequent digital subtraction angiography, demonstrating a large ruptured hepatocellular carcinoma with multiple pseudoaneurysms (arrows) and arteriovenous shunt (arrowhead); the origin of the right hepatic artery from the superior mesenteric artery noted at the CT angiography was also confirmed. (**C**) A digital subtraction angiography depicting an effective embolization of the target artery with EVOH copolymer; the onyx cast can be noted (arrow).

**Figure 3:**
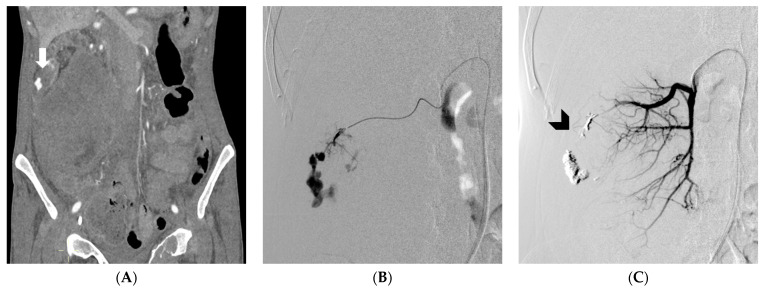
(**A**) demonstrates a CT angiography revealing spontaneous retroperitoneal bleeding caused by a ruptured pseudoaneurysm (arrow), originating from a renal tumor. (**B**) displays a digital subtraction angiography confirming the presence of a ruptured pseudoaneurysm, arising from a feeding artery of the tumor. (**C**) exhibits DSA, illustrating successful embolization, using an EVOH copolymer cast (arrowhead) (from [19], by MDPI, Basel, Switzerland, licensed under CC BY 4.0).

**Figure 4:**
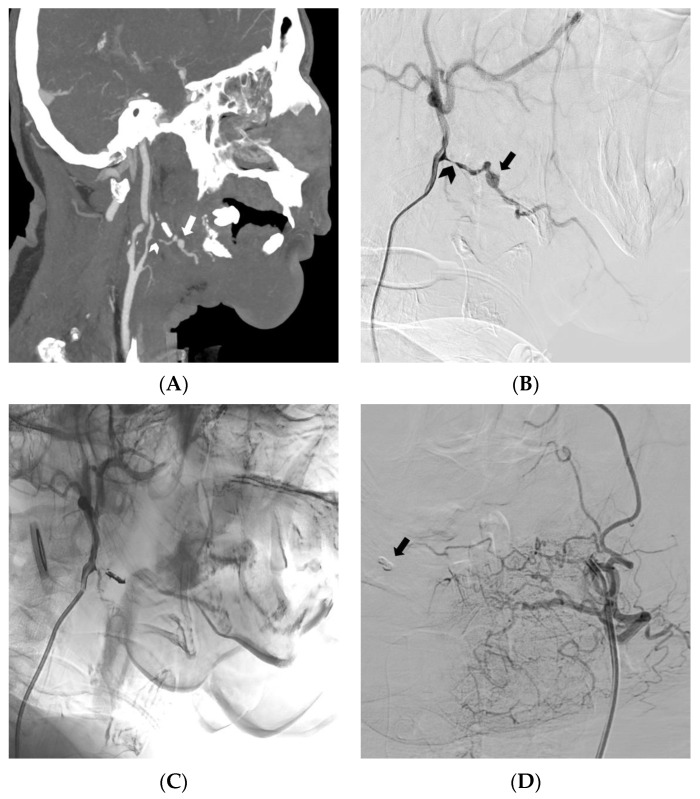
In this case of locally advanced squamous cell carcinoma of the tongue, (**A**) a CT angiography with maximum intensity projection reformatted in the sagittal plane, and (**B**) a digital subtraction angiography of the external carotid artery, were conducted. (**A**,**B**) Significant oral bleeding originated from a pseudoaneurysm (arrow) located in the context of an irregular arterial wall profile (arrowhead). (**C**) The successful embolization of the lingual artery was achieved using an EVOH copolymer, as demonstrated in the angiogram. (**D**) A contralateral digital subtraction angiography excluded retrograde bleeding, and the presence of the onyx cast can be observed (arrow) (from [19] by MDPI, Basel, Switzerland, licensed under CC BY 4.0).

This study was a multicenter (Dulbecco University Hospital, Catanzaro, Italy; Maggiore della Carità University Hospital, Novara, Italy; Circolo Hospital, Varese, Italy; Mater Domini University Hospital, Catanzaro, Italy; Pugliese-Ciaccio Hospital, Catanzaro, Italy; Cardarelli Hospital, Campobasso, Italy; San Timoteo Hospital, Termoli, Italy) analysis of the retrospectively collected data of patients with solid cancers who underwent TAE for the management of tumor hemorrhages from January 2020 to May 2023 (Figure 1, Figure 2, Figure 3 and Figure 4). The inclusion criteria were: (I) as per the Society of Interventional Radiology (SIR) standards for transcatheter arterial embolization, TAE was performed because of acute non-neurovascular hemorrhage [3]; (II) there was intratumor bleeding or bleeding into tissues locally infiltrated by the tumor itself; (III) the patient age was equal to or greater than 18 years; and (IV) there was a multidisciplinary evaluation by anesthesiologists, interventional radiologists, and surgeons. The exclusion criteria were: (I) a pregnant or breastfeeding woman; (II) in accordance with SIR recommendations, a PLT count of less than 20,000/μL and a reluctance to transfuse [18]; (III) an INR ≥ 1.8 for femoral access or INR ≥ 2.2 for radial access [18]; (IV) a hypersensitivity to the available embolic agents; (V) the hemorrhage occurring in the internal carotid artery or its associated branches; (VI) cancer-related medical conditions causing bleeding in anatomical regions other than the site of tumor growth (e.g., spontaneous retroperitoneal hematoma in cancer patients with coagulopathy); and (VII) hematologic malignancies.

A retrospective evaluation was conducted on patients who underwent transcatheter arterial embolization (TAE) for acute non-neurovascular bleeding unrelated to cancer, comprising the control group. We retrospectively analyzed consecutive patients from January 2020, to constitute the same sample size as the group of patients undergoing TAE for cancer-related bleeding. The study was performed in a retrospective fashion; therefore, no ethics committee permission was required. The research was carried out in line with the Helsinki Declaration. Before undergoing endovascular therapy, all patients signed a written informed consent form.

### 2.2. Treatment

Prior to angioembolization, a CT angiography (CTA) scan was performed, unless cases met specific criteria outlined in international recommendations or expert opinions, for which exceptions were made (e.g., upper gastrointestinal bleeding refractory to endoscopic treatment [20]). The endovascular procedure was performed in dedicated angiographic suites by an experienced interventional radiologist (with at least five years of experience). Prior to the angioembolization, a diagnostic angiography was always performed. The operator’s preference determined the embolic agent used. The embolic agent was administered under fluoroscopic supervision, in accordance with the manufacturer’s instructions. After using a non-adhesive liquid embolic agent (NALEA) or N-butyl cyanoacrylate (NBCA), the microcatheter was never reused or cleansed [19]. If there was evidence of arterial bleeding on the CTA or endoscopy, subsequently not noticed on angiography, a blind embolization was then conducted. Postembolization angiography was utilized to assess the technical success of the procedure, and identify any instances of non-target embolization. Additionally, careful consideration was given to the evaluation of potential collateral circulation based on the anatomical location of the bleeding. The anesthesiologist delivered anesthesia during the embolization, to optimize patient comfort and analgesic treatment following TAE, as appropriate. Before hospital release, and one month after TAE, participants underwent clinical evaluations and follow-up imaging.

### 2.3. Outcomes and Definitions

The rate of technical success was the primary efficacy endpoint. As a secondary efficacy endpoint, the clinical success rate was used. The complication rate was selected as the primary safety endpoint. The secondary safety outcomes were the incidence of non-target embolization, and the rate of major complications, evaluated according to the 2003 SIR classification [21].

The SIR’s reporting standards for TAE were adhered to, unless specified otherwise. (e.g., the clinical success reflects the measured results within 30 days of embolization, and is defined as the resolution of the signs or symptoms that prompted the embolization procedure) [22]. The definition of coagulopathy provided by Loffroy et al. was used [23]: INR > 1.5, partial thromboplastin time > 45 s, or PLT < 80,000/mm^3^. Bleeding on an X-ray angiography was defined by the presence of an active extravasation of the contrast medium, or a pseudoaneurysm. The 2017 SIR classification [24], the 2003 SIR classification [21], and the CIRSE classification [25] were used to classify TAE-related complications.

### 2.4. Statistical Analysis

The collected data were stored in a Microsoft Excel spreadsheet (Microsoft Inc., Redmond, WA, USA). Statistical analyses were conducted on an intention-to-treat basis, using SPSS software (SPSS, version 22 for Windows; SPSS Inc., Chicago, IL, USA) and R/R Studio software. The analyses were conducted using the Modified Intention-To-Treat population, which consisted of all the randomized participants who underwent at least one angioembolization [26,27]. The Kolmogorov–Smirnov and Shapiro–Wilk tests were employed to validate the normality assumption of the data. Categorical data are shown as a frequency (% value) [28]. Continuous data are reported as previously described [29,30]. The unpaired Student’s *t*-test [31], the chi-squared/Fisher’s exact tests, and the Wilcoxon rank-sum test [32,33] were performed as appropriate. For the tests stated above, a *p*-value of 0.05 was considered statistically significant.

## 3. Results

During the study interval (January 2020–May 2023), 92 patients underwent transcatheter arterial embolization for acute non-neurovascular cancer-related bleeding. A total of 58 (63%) procedures were performed on patients with coagulopathy. Bleeding was detected on the CT angiography in 77 (83.7%) cases. A total of 62 (67.4%) patients were on anticoagulant therapy, and 82 (89.1%) patients were on antiplatelet or anticoagulant therapy. Details are given in Table 1.

There were 92 transcatheter arterial embolizations conducted. Blind embolization was conducted in 12 (13%) cases, as no bleeding was observed using X-ray angiography (XA) (i.e., embolization of the gastroduodenal artery indicated by esophagogastroduodenoscopy findings). In all cases of the non-detection of bleeding using XA, some angiographic abnormalities (tumor neovascularization, tumor enhancement, or luminal irregularity) were, however, noted. The most common bleeding site was the liver (21.7%). Liver and lung tumors were the leading causes of bleeding. A total of 66 tumor hemorrhages were spontaneous. The most commonly used embolic agent was polyvinyl alcohol (PVA) particles (30.4%). The mean contrast volume to creatinine clearance ratio was 0.7 (±0.5). The common femoral artery was the most commonly used vascular access site (73.9%). The radiation exposure expressed by the cumulative air kerma and total dose area product was 157.2 (±59) mGy and 24.6 (±9.4) Gy/cm^2^, respectively. Table 2 contains the comprehensive procedure data.

Technical success was achieved in 82 (89.1%) cases, with the 84.8% clinical success rate related to 14 cases of rebleeding. An 83.3% technical success rate was noted. Proximal embolization was performed in 19 (20.7%) patients. Non-target embolization was detected in one case (1.1%). Complications occurred in 10 (10.9%) of the patients. VASCs (vascular access site complications) occurred at an incidence of 2.2%. According to the 2017 SIR classification for complications [24], eight (8.7%) patients experienced a minor procedure-related complication (one access site hematoma, one access site pseudoaneurysm, two post-embolization syndromes, four abscesses), and two (2.2%) patients experienced a minor procedure-related complication (one ischemic stroke, one spinal cord infarction). The 30-day bleeding-related mortality was 15.2%.

Details are given in Table 3.

There were no statistically significant differences between the group of patients undergoing TAE with liquid embolics or particles (74 patients, 80.4%) and the group of patients undergoing TAE with coils (18 patients, 19.6%) in terms of the BMI (*p* = 0.5284), INR (*p* = 0.8614), platelet count (*p* = 0.2889), D-Dimer (*p* = 0.4313), anticoagulant therapy (*p* = 0.4426), cause of bleeding (*p* = 0.7319), complications (*p* = 1), and 30-day bleeding-related mortality (*p* = 0.0777). Statistically significant differences were noted between participants undergoing TAE with liquid embolics or particles (74 patients, 80.4%) and those undergoing TAE with coils (18 patients, 19.6%) in terms of the technical success (*p* < 0.0001), clinical success (*p* < 0.0001), rate of proximal embolization (*p* = 0.0019), and 30-day rebleeding (*p* < 0.0001). Table 4 compares data from patients with and without coagulopathy.

No statistically significant differences were observed between the group of patients undergoing TAE for cancer-related bleeding, and the control group consisting of patients undergoing TAE for non-cancer-related bleeding in terms of the age (*p* = 0.1628), technical success (*p* = 0.1626), clinical success (*p* = 0.0503), 30-day rebleeding (*p* = 0.163), complications (*p* = 0.6118), and 30-day bleeding-related mortality (*p* = 1). Statistically significant differences were observed between the aforementioned groups in terms of the coagulopathy (*p* < 0.0001), and cause of bleeding (*p* < 0.0001).

Details are reported in Table 5.

## 4. Discussion

The efficacy of transcatheter arterial embolization (TAE) for the management of tumor hemorrhages in individuals diagnosed with solid cancers has been demonstrated in this multicenter retrospective cohort investigation. Technical success was achieved in 82 (89.1%) cases, with the 84.8% clinical success rate related to 14 cases of rebleeding. These findings are consistent with previous research investigating TAE in cancer-related bleeding. TAE has been recognized to be effective in a variety of bleeding cancers, including the gastrointestinal tract [11,15,34,35,36], liver [9,37,38,39], pelvis [40,41,42,43,44,45], renal [17], breast [8], lung [16,46,47], and head and neck region [48,49,50,51,52].

Park et al. reported technical and clinical success rates of 85.0% and 65.0%, respectively, in their series of 40 TAEs for gastric cancer-related gastrointestinal bleeding [35]. Gastric cancer-related bleeding accounts for about 5% of gastrointestinal bleeds [53,54], and an endoscopy may fail to recognize and stop the bleeding [55,56]. TAE is a safe and effective option when endoscopy fails or is unavailable [57,58]. Recently, the outcomes of angioembolization for the management of upper gastrointestinal hemorrhages in patients with solid malignancies were retrospectively evaluated by Gong et al., who reported a 99.1% technical success, and a 56.1% clinical success [36]. The spontaneous rupture of a hepatocellular carcinoma (HCC) occurs in up to 20% of HCC cases, and often causes a massive life-threatening hemorrhage, with a mortality rate of up to 25% [37]. In a recent case series by Nykanen et al. [39], 49 patients underwent TAE for a spontaneous hepatic tumor hemorrhage, with a 92% technical success, and a 16% 30-day rebleeding rate. In patients with uterine and bladder neoplasms, a 95% clinical success rate has been reported [41], confirming the efficacy of TAE in bleeding from pelvic tumors, as well. A recent meta-analysis reported a 95% hematuria improvement rate in patients undergoing TAE for bleeding renal cell carcinoma [17]. Up to 30% of lung cancer patients will develop hemoptysis [46]. In a retrospective review of 30 cancer patients undergoing bronchial artery embolization for the management of hemoptysis, Wang et al. observed an 86% technical success rate, and an 80% clinical success rate [16]. Technical failures are usually caused by the inability to maintain a safe and stable catheter position in the bronchial artery, failed embolization in extensive disease, or not recognizing the pulmonary artery as the site of bleeding [59]. Besides, Seki et al. have newly advocated for using transcatheter arterial chemoembolization (TACE) to treat hemoptysis in lung cancer patients [47]. In patients with bleeding head and neck cancer, a technical success of 100%, a clinical success of 82.8%, and a 30-day rebleeding rate of 17.2% were reported [51]. It should also be noted that the efficacy outcomes were in keeping with the control group of patients undergoing TAE for non-cancer-related bleeding. Hence, we can advocate for the efficacy of TAE in cancer-related bleeding, despite the lack of specific guidelines.

It is worth noting a heterogeneity in the definition of clinical success in the numerous studies on TAE [35,57,60]. It is our opinion that this evidence can lead to errors in comparing the results, and that it is desirable to use a common terminology, such as that proposed by the Society of Interventional Radiology [22]. Notably, clinical success has also been shown to be a predictor of 30-day survival after TAE [35]. Interestingly, in some cases, TAE can remove the need for emergency surgery and, subsequently, more frequent complications, providing a bridge to curative surgery for resectable cancers [61].

In the case of no evidence of active extravasation on the angiography, Tandberg et al. reported a higher rate of bleeding control (91% clinical success) when empirical embolization was performed than when conservative management was preferred [15]. In tumor-related hemorrhages, if active extravasation is not demonstrated, it is very common to still note some angiographic abnormalities, such as tumor neovascularity, tumor enhancement, and luminal irregularity [15]. Gong et al. detected positive angiographic findings (contrast extravasation and pseudoaneurysm) in only 28% of patients, and in 72% of cases they performed empiric embolizations [36]. Meehan et al. highlighted a high rate of tumor staining in cancer-related bleeding [60]. Our results support the need to look for other angiographic signs in cancer-related bleeds, in addition to active extravasation, and the opportunity to perform empirical embolization in cancer-related bleeds, especially in the upper gastrointestinal tract, the use of this practice wherein is supported by robust evidence in the scientific literature [36,58].

Within our study, transcatheter arterial embolization (TAE) exhibited a favorable safety profile when utilized for the treatment of acute tumor hemorrhages. The 30-day bleeding-related mortality was 5.4%. In their series on gastric cancer-related gastrointestinal bleeding, Park et al. reported a 12.5% bleeding-related mortality rate, with 5% minor complications (two splenic infarctions), and no major complications [35]. In a recent multicenter retrospective study of 107 patients with solid malignancies undergoing angioembolization for the management of upper gastrointestinal hemorrhages, major complications were observed in 0.9% of patients (one case of gastrointestinal perforation), and minor complications (abdominal pain, fever, and vomiting) were observed in 17.8% of patients [36]. Nykanen et al. highlighted a 31% mortality rate and a 33% major complication rate in their investigation on TAE for spontaneous hepatic tumor hemorrhage; no major complications were noted among patients with bleeding hepatic metastases, and without cirrhosis [39]. Complications occurred in 5% of TAEs performed for pelvic cancer bleeds [41]. In a retrospective review of 30 cancer patients undergoing bronchial artery embolization for the management of hemoptysis, Wang et al. observed a 6.7% minor complication rate, and a 3.3% major complication rate (one case of spinal cord infarction) [16]. Chou et al. reported a 37% hemorrhage-related mortality in a series of 63 patients undergoing TAE for massive tumor bleeding in head and neck cancer [52]. In patients with bleeding head and neck cancer, a 10% bleeding-related mortality, and a 17.5% adverse event rate were reported [51]. In addition, common or internal carotid artery embolization was performed for carotid blowout syndrome, after confirmation of collateral flow from the contralateral carotid artery, resulting in cerebral infarction in 30% of cases [51]. Theoretically, endovascular treatment with covered stents may be an ideal strategy for carotid blowout syndrome; however, adverse events such as ischemic stroke and infection are still high (10–30%) [62,63,64,65,66]. For these reasons, the ideal treatment for carotid blowout syndrome remains controversial and dependent on operator preferences [51]. Finally, in our investigation, the safety results, encompassing VASCs, demonstrate an alignment with previous studies within the realm of endovascular interventions and TAEs [67,68,69,70,71,72,73,74,75,76,77,78,79].

The best embolic agent is far from being identified, even within tumor hemorrhage embolization. Tumor biology provides some clues as to the rationale for choosing the best embolic agent. Angiogenesis is a hallmark of solid cancers [80,81,82,83,84]. Unlike the architecture of normal tissues, the tumor vasculature is abnormal, being leaky, tortuous, fragile, and blind-ended [85]. The ideal embolic agent should exclude bleeding vessels from circulation [86]. The collateral circulation depends on the anatomical region and the tumor size, and should be considered as a possible cause of rebleeding [87,88]. Thus, it can be speculated that tumor vascular bed embolization is a key factor in achieving effective TAE in bleeding cancers, preventing rebleeding episodes.

Therefore, some assessments should be taken into account when choosing the embolic agent in bleeding cancers. Firstly, bleeding vessels within the tumor vascular bed may be difficult to reach superselectively with a microcatheter. Secondly, vascular plugs, coils, and microcatheters apply a radial force to fragile vessels, thus increasing the risk of vessel wall rupture [89]. Thirdly, there may be more safety issues, as it may be challenging to deploy many coils along tortuous arteries [90,91].

For all of the above reasons, the TAE of bleeding tumors using coils might result in a higher rate of non-superselective proximal embolizations, compared to the TAE of bleeding tumors using liquid embolics or particles. Our results support this hypothesis. Furthermore, it would be appropriate to understand whether this technical difference could have clinical consequences. Our data show a trend toward a lower clinical success rate and higher rebleeding episodes for the subgroup of coil embolizations. A previous study on TAE for pelvic tumor bleeds supported our findings, showing a 6-month rebleeding rate of 60% in patients undergoing proximal embolization, compared with 14% in patients undergoing selective embolization [41]. Further studies with a prospective design and a larger sample size would be desirable, to strengthen this evidence.

Hence, multiple bleeding sites, small-caliber vessels that are challenging to catheterize, and backdoor bleeding reduce the effectiveness of coils [92,93]. Interestingly, cancer-related thrombocytopenia is not a rare condition [10], and the use of coils as the sole embolic agent, and the presence of coagulopathy have previously been noticed as independent predictors of early rebleeding [23]. Finally, proximal coil embolization may prevent further access to the tumor vascular bed, in the event of rebleeding [59]. The conditions mentioned are often encountered in the TAEs of bleeding tumors.

Conversely, some advantages of particle and liquid embolics should be noted. These embolic agents can be delivered through small-caliber arteries, reaching embolization targets significantly distant from the microcatheter tip [94,95]. Secondly, they guarantee a rapid and effective mechanical embolization, not requiring the activation of coagulation [94,95]. These features are of particular interest in the embolization of bleeding tumors, as the delivery of the embolic agent in the tumor vascular bed plays a key role in ensuring a high efficacy, and reducing the rate of rebleeding.

Despite common characteristics, there are some peculiarities that differentiate the main types of these embolic agents, which are worth summarizing. PVA particles and microspheres cause mechanical vascular occlusion, by depositing in vessels, and causing an inflammatory, and then fibrotic, reaction in the vessel wall; and they are available in different size ranges, which determine their ability to penetrate even very small vessels [96,97,98]. However, in high-flow lesions and acquired fistulas, where microspheres and particles that are too small can be shunted, larger-sized formulations are preferred; these, nevertheless, ensure more proximal embolization [91,99]. In high-flow lesions, where particles and NBCA are difficult to control, the high viscosity formulation of ethylene-vinyl alcohol (EVOH) may be preferred, to lower the risk of nontarget embolization and organ infarction [100,101]. However, the high cost may limit the use of EVOH-based NALEAs [86,90,102]. TAE with NBCA has a favorable cost-effectiveness ratio [103], but its use requires a longer learning curve than coils, and is probably less “user-friendly” than non-adhesive liquid embolic agents (NALEAs), due to the higher risk of insidious adverse events, such as the gluing of the microcatheter’s tip [104].

The limitations of this study are the retrospectivity of the analysis, the heterogeneity of the indications and tumor characteristics, the short-term follow-up, and the scarcity of data in the literature. The choice of embolic agent depended on operator preference, which could be a potential confounding factor.

## 5. Conclusions

To the best of our knowledge, this is one of the largest multicenter cohort studies to date investigating the efficacy and safety of transcatheter arterial embolization (TAE) in cancer-related bleeding.

Hence, the results of the current investigation demonstrate that transcatheter arterial embolization (TAE) is an effective, safe, and potentially life-saving option for the management of tumor hemorrhages. The TAE of bleeding tumors using coils resulted in a higher rate of non-superselective proximal embolizations, with a trend toward a lower clinical success rate and a higher number of rebleeding episodes.

Further studies are warranted in order to better understand which embolic agent is best for each type of bleeding tumor.

## Figures and Tables

**Table 1:** Population data.

Variables	All Patients (n = 92)
Age (years)	62.7 (±17.7)
Sex (M/F)	60 (65.2%)/32 (34.8%)
BMI	26 (±3.7)
eGFR (mL/min)	68 (±23)
CKD Stage	2 (1–3)
INR	1.3 (±0.3)
aPTT (s)	38.2 (±5.1)
PT (s)	14.3 (±2.8)
D-Dimer (mg/L)	1.2 (±0.7)
Fibrinogen (g/L)	1.9 (±0.6)
Platelet count (No. ×10^3^/μL)	363 (±105.6)
Coagulopathy (no/yes)	58 (63%)/34 (37%)
Hemoglobin (g/dL)	7.7 (±0.7)
CT angiography execution	80 (87%)
Bleeding on CT angiography	77 (83.7%)
Antiplatelet therapy - Single - Dual	20 (21.7%) 10 (10.9%) 10 (10.9%)
Anticoagulant therapy	62 (67.4%)
Antiplatelet AND anticoagulant therapy	0 (0%)
Antiplatelet OR anticoagulant therapy	82 (89.1%)

**Table 2:** Procedure data.

Variables	All Patients (n = 92)
Bleeding on XA	80 (87.0%)
Blind embolization	12 (13%)
Site of bleeding	
- Pelvic	12 (13.0%)
- GI	16 (17.4%)
- Liver	20 (21.7%)
- Retroperitoneal	12 (13.0%)
- Thorax	18 (19.6%)
- Head and neck	8 (8.7%)
- Limbs	6 (6.5%)
Bleeding cancer	
- Lung	16 (17.4%)
- Hepatocellular carcinoma	12 (13.0%)
- Renal cell carcinoma	10 (10.9%)
- Liver metastases	8 (8.7%)
- Tongue	8 (8.7%)
- Gastric	6 (6.5%)
- Pancreatic	6 (6.5%)
- Other	26 (28.3%)
Number of embolized vessels	1.1 (±0.3)
Cause of the bleeding	
- Spontaneous	66 (71.7%)
- Iatrogenic	18 (19.6%)
- Trauma	8 (8.7%)
Main embolic agent	
- PVA	28 (30.4%)
- Microspheres	18 (19.6%)
- Onyx or squid	16 (17.4%)
- NBCA	12 (13.0%)
- Coils	18 (19.6%)
Intraoperative unfractionated heparin (IU)	
- No	84 (91.3%)
- 2000 IU	6 (6.5%)
- 3000 IU	2 (2.2%)
Intraoperative contrast medium (mL)	36.1 (±9.5)
Volume of contrast to creatinine clearance ratio	0.7 (±0.5)
Vascular access site	
- Femoral	68 (73.9%)
- Radial	20 (21.7%)
- Brachial	4 (4.4%)
Sheath diameter, 4F/5F/6F/≥7F	10 (10.9%)/76 (82.6%)/6 (6.5%)/0 (0%)
Angiography injection technique (manual/powered)	50 (54.3%)/42 (45.7%)
CT-to-groin time (min)	51.3 (±64.4)
Procedure time (min)	28.5 (±9)
CT-to-embolization time (min)	63.2 (±79.8)
Fluoroscopy time (min)	7.4 (±2.9)
Cumulative air kerma (mGy)	157.2 (±59)
Dose area product (DAP) (Gy/cm^2^)	24.6 (±9.4)

**Table 3:** Outcome data.

Variables	All Patients (n = 92)
Technical success	82 (89.1%)
Clinical success	78 (84.8%)
Proximal embolization	19 (20.7%)
Vascular access site hemostasis - Manual compression - Vascular closure device	86 (93.5%) 6 (6.5%)
Units of packed red blood cells transfused per patient	1 (±0.6)
30-day rebleeding	14 (15.2%)
Non-target embolization	1 (1.1%)
Complications	10 (10.9%)
Vascular access site complications (VASCs)	2 (2.2%)
Complications, according to SIR classifications - None - Minor (grade 1–2) - Major (grade 3–4–5)	82 (89.1%) 8 (8.7%) 2 (2.2%)
Complications, according to CIRSE classification - Grade 0 - Grade 2 - Grade 3 - Grade 4	82 (89.1%) 4 (4.3%) 4 (4.3%) 2 (2.2%)
Treatment required for complications - None - Medical - Interventional - Surgical	82 (89.1%) 6 (6.5%) 4 (4.4%) 0 (0%)
30-day bleeding-related mortality	5 (5.4%)

**Table 4:** Comparison of data between TAE performed with liquid embolics or particles vs. TAE performed with coils.

Variables	Group 1 (n = 74) Liquid Embolics or Particles	Group 2 (n = 18) Coils	*p* Value
BMI	26.08 (±3.7)	25.78 (±3.7)	0.5284
INR	1.3 (±0.3)	1.3 (±0.3)	0.8614
D-Dimer (mg/L)	1.3 (±0.6)	1.1 (±0.7)	0.4313
Platelet count (No. ×10^3^/μL)	360.4 (±102.4)	373.8 (±120.4)	0.2889
Anticoagulant therapy	48 (64.9%)	14 (77.8%)	0.4426
Cause of the bleeding			0.7319
- Spontaneous	52 (70.3%)	14 (77.8%)	
- Other (iatrogenic or trauma)	22 (29.7%)	4 (22.2%)	
Technical success	72 (97.3%)	10 (55.6%)	<0.0001
Proximal embolization	10 (13.5%)	9 (50%)	0.0019
Clinical success	70 (94.6%)	8 (44.4%)	<0.0001
30-day rebleeding	4 (5.4%)	10 (55.6%)	<0.0001
Complications	8 (10.8%)	2 (11.1%)	1
30-day bleeding-related mortality	2 (2.7%)	3 (16.7%)	0.0777

**Table 5:** Comparison of data between TAE performed for cancer-related bleeding, and a control group consisting of TAE performed for non-cancer-related bleeding.

Variables	Group 1 (n = 92) Cancer-Related Bleeding	Group 2 (n = 92) Non-Cancer-Related Bleeding	*p* Value
Age (years)	62.7 (±17.7)	58 (±19.7)	0.1628
Coagulopathy	34 (37%)	13 (14.1%)	<0.0001
Cause of the bleeding - Spontaneous - Other (iatrogenic or trauma)	66 (71.7%) 26 (28.3%)	11 (11.9%) 81 (88.1%)	<0.0001
Technical success	82 (89.1%)	88 (95.6%)	0.1626
Clinical success	78 (84.8%)	87 (94.6%)	0.0503
30-day rebleeding	14 (15.2%)	7 (7.6%)	0.163
Complications	10 (10.9%)	7 (7.6%)	0.6118
30-day bleeding-related mortality	5 (5.4%)	4 (4.3%)	1

## Data Availability

The data presented in this study are available on request from the corresponding author. The data are not publicly available, due to privacy issues.

## Abbreviations

APTT, activated partial thromboplastin time; CKD, chronic kidney disease; CTA, CT angiography; DAP, dose-area product; eGFR, estimated glomerular filtration rate; EVOH, ethylene-vinyl alcohol; HCC, hepatocellular carcinoma; INR, international normalized ratio; NALEA, non-adhesive liquid embolic agent; NBCA, N-butyl cyanoacrylate; PT, prothrombin time; PVA, polyvinyl alcohol; TACE, transcatheter arterial chemoembolization; TAE, transcatheter arterial embolization; VASCs, vascular access site complications; XA, X-ray angiography.
